# Characterization of the off-flavor from *Pichia pastoris* GS115 during the overexpression of an α-l-rhamnosidase

**DOI:** 10.1093/jimb/kuad035

**Published:** 2023-11-06

**Authors:** YuXuan Yao, ShengLan Zheng, ShiLin Chi, Feng Chen, Ning Cai, ZhenZhen Cai, Zhipeng Li, Hui Ni

**Affiliations:** College of Ocean Food and Biological Engineering, Jimei University, Xiamen 361021, People's Republic of China; College of Ocean Food and Biological Engineering, Jimei University, Xiamen 361021, People's Republic of China; College of Ocean Food and Biological Engineering, Jimei University, Xiamen 361021, People's Republic of China; Department of Food, Nutrition and Packaging Sciences, Clemson University, Clemson, SC 29634, USA; Xiamen Ocean Vocational College, Xiamen, Fujian 361021, People's Republic of China; Xiamen Ocean Vocational College, Xiamen, Fujian 361021, People's Republic of China; College of Ocean Food and Biological Engineering, Jimei University, Xiamen 361021, People's Republic of China; Key Laboratory of Food Microbiology and Enzyme Engineering Technology of Fujian Province, Xiamen, Fujian 361021, People's Republic of China; Research Center of Food Biotechnology of Xiamen City, Xiamen, Fujian 361021, People's Republic of China; College of Ocean Food and Biological Engineering, Jimei University, Xiamen 361021, People's Republic of China; Xiamen Ocean Vocational College, Xiamen, Fujian 361021, People's Republic of China; Key Laboratory of Food Microbiology and Enzyme Engineering Technology of Fujian Province, Xiamen, Fujian 361021, People's Republic of China; Research Center of Food Biotechnology of Xiamen City, Xiamen, Fujian 361021, People's Republic of China

**Keywords:** *Pichia pastoris*, Off-flavor, GC–MS analysis

## Abstract

The off-flavor of *Pichia pastoris* strains is a negative characteristic of proteins overexpressed with this yeast. In the present study, *P. pastoris* GS115 overexpressing an α-l-rhamnosidase was taken as the example to characterize the off-flavor via sensory evaluation, gas chromatography–mass spectrometer, gas chromatography–olfaction, and omission test. The result showed that the off-flavor was due to the strong sweaty note, and moderate metallic and plastic notes. Four volatile compounds, that is, tetramethylpyrazine, 2,4-di-*tert*-butylphenol, isovaleric acid, and 2-methylbutyric acid, were identified to be major contributors to the sweaty note. Dodecanol and 2-acetylbutyrolactone were identified to be contributors to the metallic and plastic notes, respectively. It is the first study on the off-flavor of *P. pastoris* strains, helping understand metabolites with off-flavor of this yeast. Interestingly, it is the first study illustrating 2-acetylbutyrolactone and dodecanol with plastic and metallic notes, providing new information about the aromatic contributors of biological products.

**Importance:**

The methylotrophic yeast *Pichia pastoris* is an important host for the industrial expression of functional proteins. In our previous studies, *P. pastoris* strains have been sniffed with a strong off-flavor during the overexpression of various functional proteins, limiting the application of these proteins. Although many yeast strains have been reported with off-flavor, no attention has been paid to characterize the off-flavor in *P. pastoris* so far. Considering that *P. pastoris* has advantages over other established expression systems of functional proteins, it is of interest to identify the compounds with off-flavor synthesized in the overexpression of functional proteins with *P. pastoris* strains. In this study, the off-flavor synthesized from *P. pastoris* GS115 was characterized during the overexpression of an α-l-rhamnosidase, which helps understand the aromatic metabolites with off-flavor of *P. pastoris* strains. In addition, 2-acetylbutyrolactone and dodecanol were newly revealed with plastic and metallic notes, enriching the aromatic contributors of biological products. Thus, this study is important for understanding the metabolites with off-flavor of *P. pastoris* strains and other organisms, providing important knowledge to improve the flavor of products yielding with *P. pastoris* strains and other organisms.

**One-Sentence Summary:**

Characterize the sensory and chemical profile of the off-flavor produced by one strain of P. pastoris in vitro.

## Graphical Abstract

**Graphical Abstract:**
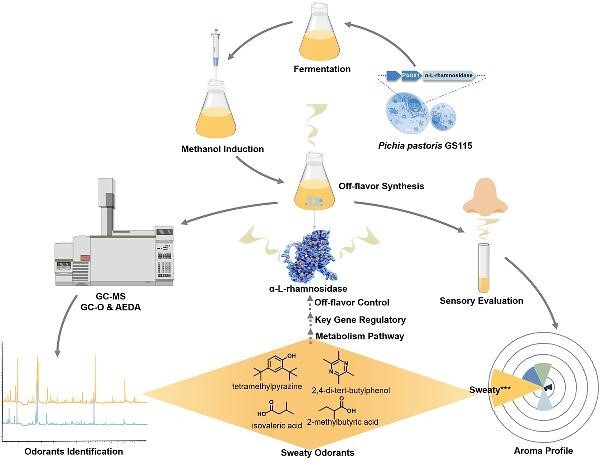
The strong off-flavor (sweaty odor) was identified as a contribution of tetramethylpyrazine, 2,4-di-tert-butylphenol, isovaleric acid, and 2-methylbutyric acid.

## Introduction

The methylotrophic yeast *Pichia pastoris* is an important strain used as an industrial strain. *Pichia pastoris* strains can achieve a very high cell density and have a strong, tightly controlled, methanol-inducible promoter. In most cases, the host *P. pastoris* offers advantages over other established expression systems of functional proteins (Ahmad et al., 2014). Hitherto, there are more than 4.4 million international patents involving the use of *P. pastoris* as a host system to overexpress up to 5000 kinds of proteins (Fischer & Glieder, 2019), including α-l-rhamnosidase (Li et al., 2019), endoxylanase (Wang et al., 2016), and tannase (Lebesi & Tzia, 2012).

Recently, *P. pastoris* strains have been observed to synthesize a strong off-flavor during the overexpression of various functional proteins such as α-l-rhamnosidase (Li et al., 2020a), β-d-glucosidase (Ni et al., 2021), and β-xylosidase (Zhang et al., 2020a), limiting the application of these proteins in food, nutraceutical, and pharmaceutical industries. Although many yeast strains have been reported with off-flavor (Wang et al., 2020), little attention has been paid to the off-flavor in *P. pastoris* so far. Therefore, it is of interest to identify the compounds with off-flavor synthesized in the overexpression of functional proteins with *P. pastoris* strains.

Currently, various techniques and procedures are available for off-flavor analysis. The analysis using solid-phase microextraction combined with gas chromatography–mass spectrometry–olfactometry (GC–MS–O) shows that short-chain fatty acids, organic acids, higher alcohols, and esters are the potential compounds for the off-odor of *Brettanomyces bruxellensis* in red wines (Fugelsang & Zoecklein, 2003). GC–MS analysis indicates that isobutyric acid, isovaleric acid, and β-phenylethanol contribute to the unpleasant odor of the soy sauce produced by *Zygosaccharomyces rouxii* (Tomita & Yamamoto, 1997). Solvent-assisted flavor evaporation coupled with GC–MS–O analysis indicates that 4-methylphenol, 3-methylpyridine, 3-methylbutanoic acid, and propionic acid are the main contributors to the off-flavors of Angel yeast (Zhang et al., 2017). In addition, the aroma extract dilution analysis (AEDA) is the prevailing method for identifying key odor compounds via GC–MS–O analysis (Grosch, 1993). These studies provide multimethod references for identifying the off-flavor compounds of *P. pastoris* strains.

α-l-Rhamnosidases that specifically hydrolyze the terminal α-l-rhamnosyl-linkages have extensive applications in food and pharmaceutical industries, including improving the aroma of wine (Spagna et al., 2000) and juice (Busto et al., 2007). In our previous studies, α-l-rhamnosidases from various resources were overexpressed with *P. pastoris* strains such as GS115 and SMD1168 (Li et al., 2019, 2020b; Liao et al., 2019). During these studies, *P. pastoris* strains were observed to synthesize a strong off-flavor. In the context that the compounds with the off-flavor are unclear for *P. pastoris* during the overexpression of proteins, the present study aims to characterize the off-flavor synthesized in *P. pastoris* GS115 during the overexpression of the α-l-rhamnosidase from *Aspergillus tubingensis*, which may help understand metabolites with off-flavor in *P. pastoris* strains and other biological processes.

## Materials and Methods

### Reagents and Chemicals

All chemical standards and internal standards were obtained commercially at high-purity grade (GC grade). Among them, dichloromethane, 2,4,6-trimethylpyridine, benzyl alcohol, decyl aldehyde, dodecyl aldehyde, 2,4-di-*tert*-butylphenol, and *n*-alkanes C_7_–C_30_ were obtained from Sigma–Aldrich Co., Ltd (Shanghai, China); nonanal, benzaldehyde, limonene, and phenethyl alcohol were obtained from Shanghai Aladdin Co., Ltd (Shanghai, China); ethanol, pantolactone, decanoic acid, dodecanoic acid, 3-methyl-1-butanol, 1-methylpropyl acetate, isobutyric acid, ethyl butyrate, *m*-xylene, isovaleric acid, *o*-xylene, 2-methyl butyric acid, 2,5-dimethylpyrazine, 2,6-dimethylpyrazine, δ-valerolactone, 3-methylthiopropanol, trimethylpyrazine, tetramethylpyrazine, methyl phenylacetate, benzothiazole, nonanoic acid, 2-acetylbutyrolactone, geranylacetone, dimethyl phthalate, undecenoic acid, dodecanol, 2-tridecanone, 2,2,4-trimethyl-1,3-pentanediol diisobutyrate, and 2-pentadecanone were obtained from Shanghai Macklin Biochemical Co., Ltd (Shanghai, China). Tryptone, yeast extract powder, and agar were purchased from Huankai Microbial Co., Ltd (Guangdong, China); yeast nitrogen base (YNB) and biotin B were purchased from Beijing Solarbio Co., Ltd (Beijing, China). Anhydrous sodium sulfate (Na_2_SO_4_), phosphate buffer (NaH_2_PO_4_, Na_2_HPO_4_), glycerol, glucose, and methanol were obtained from Sinopharm Chemical Reagent Co., Ltd (Shanghai, China).

### Strain and Fermentation

*Pichia pastoris* (*Komagataella phaffii*) GS115 (his4) was purchased from Invitrogen Co., Ltd (Guangzhou, China). The α-l-rhamnosidase gene was cloned from *A. tubingensis*. The methanol-inducible fermentation was conducted following the protocol in a previous study (Li et al., 2018). In short, the recombinant *P. pastoris* was first activated in 30 mL Yeast Extract Peptone Dextrose Medium medium (containing 10 g/L yeast extract powder, 20 g/L peptone, and 20 g/L glucose) for 16 hr, and then was shifted to 100 mL Buffered Glycerol-complex Medium (BMGY) medium (YNB 13.4 g/L, yeast extract powder 14.0 g/L, peptone 28.0 g/L, 0.1 M potassium phosphate, biotin 0.4% w/w, glycerol 1% w/w, pH = 6.0) for 18 hr. Then, the cells were harvested by centrifugation and shifted to Buffered Methanol-complex Medium (BMMY) medium (similar to BMGY except for the substitution of 0.5% MeOH for glycerol) for cell growth and enzyme expression for 7 days. For induction of the protein expression, methanol was injected at a ratio of 0.5% (v/v) every 24 hr. Based on our primary experiments with sensory evaluation and GC–MS analysis, the blank BMMY medium, the medium added with methanol (0.5%) before fermentation (MAM), was almost odorless and contained 22 volatile compounds, including esters, acids, alcohols, and aldehydes. The fermented broth with BMMY medium before methanol induction (BMI) was similarly odorless and contained 23 volatile compounds that are mainly esters, acids, aldehydes, pyrazines, and ketones. The concentration of volatile compounds in both MAM and BMI was very low. The detailed volatile constituents in MAM, BMI, and fermented broth after methanol induction (AMI) are shown in Table 1.

**Table 1.** Volatile Compounds of the Samples Methanol-Added Medium (MAM), Before Methanol Induction (BMI), and After Methanol Induction (AMI).

		Aroma			Characteristic ion	Dentification	Standard		Rang	MAM	BMI	AMI
No.	RT^a^	compound	RI^b^	RI^c^	fragment	basis^d^	curves	*R* ^2^	(mg/L)	(mg/L)	(mg/L)	(mg/L)
Alcohols												
1	4.678	3-Methyl-1-butanol	743	743	42 70 84	MS, Std, RI	*Y* = 0.2957X−0.0081	0.9999	0.03–10	-	-	6.22 ± 0.12
2	11.801	Benzyl alcohol	1039	1038	79 108 51	MS, Std, RI	*Y* = 0.4606X−0.2043	0.9970	0.03–30	-	-	36.62 ± 0.19
3	13.507	Phenethyl alcohol	1116	1116	91 122 65	MS, Std, RI	*Y* = 0.8985X−0.5400	0.9976	0.03–30	4.32 ± 0.06	-	65.16 ± 0.28
4	20.679	Dodecanol	1490	1490	41 69 93	MS, Std, RI	*Y* = 0.8452X−0.1138	0.9992	0.03–80	-	-	2146.29 ± 7.22
Esters												
1	5.146	1-Methylpropyl acetate	763	-	43 87 61	MS, Std	*Y* = 1.3443X−0.0037	0.9999	0.03–10	0.09 ± 0.05	-	0.44 ± 0.01
2	6.116	Ethylbutyrate	805	805	43 71 88	MS, Std, RI	*Y* = 0.4014X−0.0044	0.9999	0.03–10	-	-	3.32 ± 0.33
3	9.964	δ-Valerolactone	961	961	42 100 73	MS, Std, RI	*Y* = 0.2952X−0.0127	0.9997	0.03–10	-	2.37 ± 0.26	6.67 ± 0.77
4	11.857	Pantolactone	1042	1032	71 43 85	MS, Std, RI	*Y* = 0.3612X−0.2335	0.9995	0.03–80	6.06 ± 0.93	-	-
5	14.814	Methyl phenylacetate	1178	1178	91 150 39	MS, Std, RI	*Y* = 1.4740X−0.0316	0.9999	0.03–10	-	-	8.13 ± 0.2
6	17.471	2-Acetylbutyrolactone	1312	1312	43 86 60	MS, Std, RI	*Y* = 0.1051X−0.0133	0.9943	0.03–10	-	89.62 ± 2.69	95.82 ± 1.72
7	20.041	Dimethyl phthalate	1454	1454	163 77 133	MS, Std, RI	*Y* = 1.2893X−0.0244	0.9999	0.03–10	0.81 ± 0.56	2.91 ± 0.08	6.45 ± 0.17
8	22.355	2,2,4-Trimethyl-1,3-pentanediol diisobutyrate	1591	1588	55 69 82	MS, Std, RI	*Y* = 1.6715X−0.0323	0.9997	0.03–10	-	1.46 ± 0.01	-
Acids												
1	5.578	Isobutyric acid	765	765	43 73 88	MS, Std, RI	*Y* = 0.2883X−0.0582	0.9991	0.03–30	11.49 ± 0.07	-	55.35 ± 1.06
2	6.295	Butanoic acid	812	812	60 41 88	MS, RI	SCIS	-	-	4.34 ± 0.16	-	-
3	8.181	Isovaleric acid	888	888	60 43 87	MS, Std, RI	*Y* = 0.8888X−0.0256	0.9999	0.03–80	6.72 ± 0.03	-	303.39 ± 1.72
4	8.470	2-Methylbutyric acid	899	898	74 57 87	MS, Std, RI	*Y* = 0.9277X−0.0931	0.9991	0.03–80	2.98 ± 0.12	-	187.6 ± 1.4
5	12.550	Levulinic acid	1073	1063	43 85 73	MS, RI	SCIS	-	-	10.29 ± 0.16	-	-
6	16.521	Phenylacetic acid	1263	1263	91 136 65	MS, RI	SCIS	-	-	2.94 ± 0.71	4.19 ± 2.46	50.2 ± 1.7
7	16.735	Nonanoic acid	1274	1274	60 57 115	MS, Std, RI	*Y* = 0.4465X−0.3004	0.9951	0.03–30	4.81 ± 0.13	-	36.23 ± 0.75
8	18.070	3-Phenylpropionic acid	1344	1344	91 150 78	MS, Std, RI	*Y* = 0.2351X−0.0247	0.9991	0.03–80	0.79 ± 0.07	-	1.24 ± 0.9
9	18.437	3-Decanoic acid	1364	1364	60 41 91	MS, Std, RI	*Y* = 0.2003X−0.7213	0.9999	0.03–80	21.23 ± 0.25	-	-
10	20.548	Undecenoic acid	1483	1484	56 69 98	MS, Std, RI	*Y* = 0.6127X−0.1023	0.9995	0.03–80	-	1029.71 ± 11.6	-
11	21.825	Dodecanoic acid	1559	1559	73 43 85	MS, Std,RI	*Y* = 0.1708X−0.9701	0.9999	0.03–80	29.02 ± 0.73	-	-
12	21.871	Cis-5-Dodecenoic acid	1562	1562	55 69 82	MS, RI	SCIS	-	-	-	6.76 ± 0.28	-
13	25.242	Myristic acid	1765	1765	70 97 41	MS, RI	SCIS	-	-	-	-	103.97 ± 0.49
14	30.525	Palmitic acid	1980	1980	73 43 129	MS, RI	SCIS	-	-	-	-	1112.75 ± 8.85
Aldehydes												
1	8.659	3-(Methylthio)propionaldehyde	907	907	48 104 76	MS, RI	SCIS	-	-	3.01 ± 1.44	**-**	**-**
2	10.055	Benzaldehyde	965	965	77 106 51	MS, Std, RI	*Y* = 0.4718X−0.0124	0.9999	0.03–10	2.33 ± 0.41	7.35 ± 0.29	10.08 ± 0.26
3	13.286	Nonanal	1106	1106	49 84 70	MS, Std, RI	*Y* = 0.3961X−0.0073	0.9999	0.03–10	1.93 ± 0.07	4.73 ± 0.16	7.06 ± 0.38
4	15.425	Decyl aldehyde	1207	1207	57 82 68	MS, Std, RI	*Y* = 0.1529X−0.0040	0.9998	0.03–10	2.77 ± 0.21	4.98 ± 0.63	15.73 ± 2.22
5	18.970	Dodecyl aldehyde	1393	1392	45 82 70	MS, Std, RI	*Y* = 0.1876X−0.0058	0.9999	0.03–10	-	9.42 ± 0.54	-
Pyrazines												
1	8.813	2,5-Dimethylpyrazine	913	913	108 42 81	MS, Std, RI	*Y* = 0.6836X−0.0159	0.9999	0.03–10	-	32.92 ± 0.1	-
2	8.868	2,6-Dimethylpyrazine	916	916	108 42 81	MS, Std, RI	*Y* = 0.5896X−0.0157	0.9999	0.03–10	-	-	43.6 ± 1.41
3	10.993	Trimethylpyrazine	1004	1004	42 122 81	MS, Std, RI	*Y* = 0.8209X−0.0136	0.9990	0.03–10	-	18.67 ± 0.4	32.28 ± 0.5
4	12.864	Tetramethylpyrazine	1086	1086	136 54 95	MS, Std, RI	*Y* = 0.3783X−0.0015	0.9999	0.03–10	-	4.98 ± 0.53	33.44 ± 0.65
Ketones												
1	19.975	Geranylacetone	1450	1450	43 69 107	MS, Std, RI	*Y* = 0.4218X−0.0128	0.9998	0.03–10	-	2.52 ± 0.36	-
2	20.775	2-Tridecanone	1496	1496	58 71 85	MS, Std, RI	*Y* = 0.8079X−0.0204	0.9997	0.03–10	-	0.9 ± 0.01	-
3	24.110	2-Pentadecanone	1701	1702	58 71 85	MS, Std, RI	*Y* = 0.5843X−0.0150	0.9998	0.03–10	-	7.68 ± 0.11	39.07 ± 0.94
Sulfur compounds												
1	10.496	3-Methylthiopropanol	983	983	106 281 61	MS, Std, RI	*Y* = 0.2957X−0.0081	0.9977	0.03–30	-	-	38.1 ± 1.37
2	15.976	Benzothiazole	1235	1236	135 108 69	MS, Std, RI	*Y* = 0.7465X−0.0230	0.9998	0.03–10	-	1.18 ± 0.05	3.16 ± 0.06
Amides												
1	9.063	Isobutyramide	924	923	44 72 87	MS, RI	SCIS	-	-	-	-	5.23 ± 0.45
2	19.208	2-Phenylacetamide	1406	1411	92 135 65	MS, RI	SCIS	-	-	-	2.38 ± 0.22	-
Benzodiazepines												
1	7.772	m-Xylene	871	870	91 106 77	MS, Std, RI	*Y* = 0.9455X−0.0025	0.9999	0.03–10	-	1.51 ± 0.09	-
2	8.315	o-Xylene	893	893	91 106 51	MS, Std, RI	*Y* = 1.0967X−0.0034	0.9998	0.03–10	0.36 ± 0.13	0.83 ± 0.12	-
Phenol												
1	13.409	Maltol	1111	1111	126 43 71	MS, RI	SCIS	-	-	1.82 ± 0.99	-	-
2	21.082	2,4-Di-*tert*-butylphenol	1514	1513	191 57 206	MS, Std, RI	*Y* = 1.4646X−0.0717	0.9998	0.03–30	6.93 ± 0.06	16.96 ± 0.34	82.26 ± 2.68
Olefin												
1	11.643	Limonene	1032	1032	68 93 41	MS, Std, RI	*Y* = 0.2852X−0.0001	0.9999	0.03–10	1.21 ± 0.04	12.35 ± 0.12	5.54 ± 0.31

^a^Linear retention time calculated on Rtx-5 MS column.

^b^Linear retention index calculated on Rtx-5 MS column.

^c^Linear retention index reported from http://webbook.nist.gov/chemistry/.

^d^Method of identification: MS, compounds were identified by matching mass spectrometry spectra in the data library (NIST11, NIST11s, and FNSSC 1.3); Std, compound confirmed by matching standard reference; and when only MS or RI is available for the identification of a compound, it must be considered as an attempt of identification.

- = not available; SCIS = concentration of compound estimated by internal standard.

### Sensory Analysis

The samples were sensorially evaluated in random order. According to previous research works (Lin et al., 2014; Zhang et al., 2017; Zheng et al., 2020), eight aroma descriptors, that is, sweaty, roasted, metallic, deep fried/fatty, green, honey-like, floral, and plastic, were sensorially evaluated. A 12-member sensory evaluation panel (3 males and 9 females, 20–30 years old) was trained once per day for 4 weeks before the evaluation. The panelists were asked to determine the intensity of these descriptors by rating scores between 0 and 5, where 0 is unsniffed and 5 is very strong intensity.

### GC–MS Analysis

The 70 mL suspensions were fetched from the fermented broth AMI with centrifuge, and were extracted for 12 h with 105 mL dichloromethane. The organic phase (lower phase) was separated into a separatory funnel, followed by concentration with a termovap sample concentrator. Anhydrous sodium sulfate was added into the concentrated extract to remove water. The final extract was condensed to 1 mL. For the analysis using GC–MS, 200 μL of the condensed extract was mixed with 300 μL dichloromethane and 1 μL 1000 ppm 2,4,6-trimethylpyridine (dissolved in ethanol) as internal standards.

A QP2020 GC–MS (Shimadzu, Kyoto, Japan) was utilized for both qualitative and quantitative analyses. Full-scan mode was utilized for qualitative analysis, while SIM mode was utilized for quantitative analysis. One microliter of sample was injected in the splitless mode at 250°C. Chromatographic separation was achieved using a 30 m × 0.25 mm × 0.25 μm Rtx-5MS column (Bellefonte, PA, USA) at a flow rate of 3.13 mL/min with the carrier gas helium (99.999% purity). The GC oven temperature was maintained at 35°C for 3 min, ramped to 200°C at a rate of 8°C/min, maintained for 8 min, ramped again to 270°C at a rate of 5°C per minute, and then maintained for 10 min. The MS analysis was operated in electron impact mode at 70 eV with the scanning range from 35 to 500 m/z. The solvent delay was 3 min. The transfer line and ion source temperatures were 250 and 230°C, respectively.

The reference databases (NIST11, NIST11s, and FFNSC1.3) were used to search and screen similar substances with mass spectrometry matching greater than 80%. Moreover, retention index (RI) was calculated from each compound, based on analyses of *n-*alkane (C_7_–C_30_) following Vandendool and Kratz RI for using a temperature ramp (Vandendool & Kratz, 1963). Thereafter, most of the volatile compounds were identified by comparing their detected mass spectra and RI with those of standard compounds. The 3-methyl-1-butanol, 1-methylpropyl acetate, isobutyric acid, ethyl butyrate, m-xylene, isovaleric acid, o-xylene, 2-methylbutyric acid, 2,5-dimethylpyrazine, 2,6-dimethylpyrazine, δ-valerolactarone, benzaldehyde, 3-methylthiopropanol, trimethylpyrazine, limonene, benzyl alcohol, tetramethylpyrazine, nonanal, phenethyl alcohol, methyl phenylacetate, decyl aldehyde, benzothiazole, nonanoic acid, 2-acetylbutyrolactone, dodecyl aldehyde, geranylacetone, dimethyl phthalate, undecenoic acid, dodecanol, 2-tridecanone, 2,4-di-*tert*-butylphenol, 2,2,4-trimethyl-1,3-pentanediol diisobutyrate, pantolactone, decanoic acid, dodecanoic acid, 3-phenylpropionic acid, and 2-pentadecanone were quantitatively analyzed in the SIM model by an external standard method according to their respective calibration curves. The content of volatiles without matched standards, including isobutyramide, phenylacetic acid, 2-phenylacetamide, cis-5-dodecenoic acid, myristic acid, butanoic acid, 3-(methylthio) propionaldehyde, levulinic acid, maltol, and palmitic acid, was tentatively analyzed using the internal standard method with 2,4,6-trimethylpyridine as the internal standard.

### GC–MS–O Analysis

A QP 2020 GC–MS (Shimadzu, Kyoto, Japan) equipped with an OP 275 Olfactory Detector Port (GL Sciences Inc., Kyoto, Japan) was used. The sample was separated on a 30 m × 0.25 mm × 0.25 μm Rtx-5 MS fused silica capillary column (Bellefonte, PA, USA). The operating conditions for GC–MS–O were the same as for the GC–MS. The volatile extract was split between the olfactory detection port and MS with 16:9 proportions. The transfer line to the GC-O sniffing port was held at 200°C; humidified air was added to the sniffing port at 50 mL/min to maintain olfactory sensitivity by reducing dehydration of mucous membranes in the nasal cavity (Ni et al., 2021). AEDA analysis was conducted by the dilution of 1, 2, 4, 8, 16, 64, and 1024 folds. The aroma compounds were identified by comparing their MS fragments and RIs with those of authentic standards. The flavor dilution (FD) factor was defined as the maximum dilution where the aroma compound could be detected (Fan et al., 2015). Each diluted sample was analyzed consecutively three times by three panelists.

### Aroma Recombination and Omission Tests

The recombination and omission experiments were conducted according to the method mentioned in the previous study (Neugebauer et al., 2020). Based on aromatic compounds in the GC–MS–O analysis, 13 key volatile compounds, that is, isovaleric acid, 2-methylbutyric acid, 2,6-dimethylpyrazine, trimethylpyrazine, tetramethylpyrazine, nonanal, phenethyl alcohol, methyl phenylacetate, 2-acetylbutyrolactone, 3-phenylpropionic acid, dimethyl phthalate, dodecanol, and 2,4-di-*tert*-butylphenol were chosen to simulate the AMI sample with the sterilized BMMY medium as the substrate. All the compounds were tested in the concentration detected in the samples. To further analyze the contribution of each volatile, single-omission models were constructed by omitting the volatile compounds one by one from the AMI model.

### Statistical Analysis

Each sample was prepared and analyzed three times. The average and standard deviation were calculated with the Office 2019 software. The radar chart was drawn with the Office 2019 software. Significant analysis (Least Significant Difference) was conducted by the IBM SPSS 26.0 software.

## Results

### Descriptive Sensory Evaluation of the Off-Flavor

**Fig. 1.**
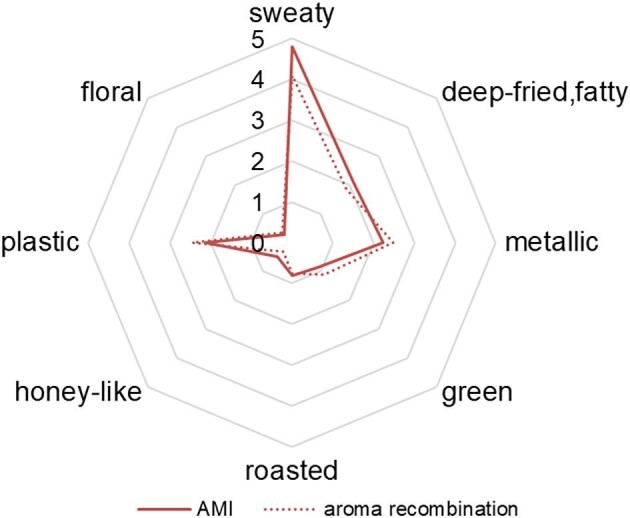
Aroma profiles and aroma recombination model of the sample after methanol induction (AMI).

To preliminarily evaluate the off-flavor, the fermented broth AMI was taken for the sensory evaluation. Methanol induction is a critical approach for promoting the heterologous expression of proteins. As shown in Fig. 1, the AMI sample was sniffed to have a strong sweaty note (score = 4.80), a moderate metallic note (score = 2.24), and a moderate plastic note (score = 2.13). A researcher has reported that yeast extract was dominated by fermented, caramel, and roasted notes (Alim et al., 2018). Another researcher regarded “sweaty” note as similar to “rancid” and “sour” notes in the case of describing the off-flavor of foods (Rudman et al., 2018). Therefore, the off-flavor might be attributed to the strong sweaty note and moderate metallic and plastic notes.

### Qualitative and Quantitative Analysis of Volatile Content

For identifying the volatile compounds contributing to the off-flavor, the AMI was submitted to GC–MS analysis. According to Table 1, a total of 30 volatile compounds were detected in the AMI, including eight acids, six esters, one ketone, four alcohols, three aldehydes, three pyrazines, two sulfur compounds, one olefin, one amide, and one phenol. The quantitative analysis showed that the AMI had a noticeable content of alcohol and acid compounds, including dodecanol (2146.29 mg/L), isovaleric acid (303.39 mg/L), 2-methylbutyric acid (187.6 mg/L), palmitic acid (1112.75 mg/L), and myristic acid (103.97 mg/L) (Table 2). A previous study has shown that *Saccharomyces cerevisiae* can synthesize various aromatic volatiles, including higher alcohols, fatty acids, acetates, ethyl esters, ketones, and aldehydes (Chen & Xu, 2010). In general, the results of the present study seem to be similar to those of the previous study in that *P. pastoris* can synthesize aromatic higher alcohols, fatty acids, acetates, ethyl esters, ketones, and aldehydes.

**Table 2.** Aroma Extract Dilution Analysis of Before Methanol Induction (BMI) and After Methanol Induction (AMI) Samples

No.	Odorants	Odor characteristic^a^	RI^b^	FD factor^c^
Sweaty				
1	3-Methyl-1-butanol	Sour, herb	743	1
2	Isobutyric acid	Sour, leather-like, gasoline	781	1
3	Ethyl butyrate	Sour, bitter	804	1
4	Isovaleric acid	Sweaty, rancid, sour	887	128
5	2-Methylbutyric acid	Sweaty, rancid, sour	898	128
6	Benzaldehyde	Sour, paint	964	1
7	3-Methylthiopropanol	Sour, paint	981	1
8	Benzyl alcohol	Sour, bitter	1038	1
9	Tetramethylpyrazine	Rancid, moldy, fermented	1085	256
10	Nonanal	Sweaty, sour	1104	4
11	Methyl phenylacetate	Sour, leather-like	1176	2
12	3-Phenylpropionic acid	Sour	1344	512
13	3-Decenoic acid	Sour	1363	1
14	2,4-Di-*tert*-butylphenol	Sour, phenol	1510	256
Metallic				
1	Dimethyl phthalate	Metallic, rust, bitter	1451	2
2	Dodecanol	Metallic, rust, bitter	1489	256
Plastic				
1	2-Acetylbutyrolactone	Plastic, rubbery	1312	2
Roasted				
1	Trimethylpyrazine	Roasted, moldy	1002	4
Floral				
1	Phenethyl alcohol	Rosy, fermented	1116	16
Deep-fried, fatty				
1	2,6-Dimethylpyrazine	Fatty, salty	915	128

^a^Odor descriptions perceived by panelists through the sniffing port.

^b^Linear retention index calculated on Rtx-5 MS column.

^c^Flavor dilution perceived through the sniffing port.

### GC-O Analysis

The GC-O analysis showed that 14 compounds, that is, 3-methyl-1-butanol, isobutyric acid, ethyl butyrate, isovaleric acid, 2-methylbutyric acid, benzaldehyde, 3-methylthiopropanol, benzyl alcohol, tetramethylpyrazine, nonanal, methyl phenylacetate, 3-phenylpropionic acid, 3-decenoic acid, and 2,4-di-*tert*-butylphenol, were sniffed with a sweaty/rancid/sour note. Dimethyl phthalate and dodecanol were sniffed with metallic notes. Limonene and 2-pyrrolidinone were sniffed with fragrant notes. The phenethyl alcohol, 2-acetylbutyrolactone, trimethylpyrazine, and 2,6-dimethylpyrazine were sniffed with floral, plastic, roasted, and deep fried/fatty notes, respectively. It has been reported that yeasts generally synthesized δ-valerolactone, dimethyl phthalate, phenylacetic acid, benzaldehyde, nonanal, decyl aldehyde, trimethylpyrazine, tetramethylpyrazine, benzothiazole, 2,4-di-*tert*-butylphenol, and limonene as the basic metabolic products with “generalist” in aroma (Ames & Leod, 2006; Comuzzo et al., 2006). It seems that the *P. pastoris* strain and other yeasts can similarly synthesize sniffed benzaldehyde, nonanal, tetramethylpyrazine, 2,4-di-*tert*-butylphenol, etc. However, the *P. pastoris* strain synthesized high FD of isovaleric acid, 2-methyl butyric acid, tetramethylpyrazine, 3-phenylpropionic acid, 2,4-di-*tert*-butylphenol, dodecanol, and 2,6-dimethyl pyrazine, whereas other yeast had not been reported to synthesize these compounds.

### Recombination and Omission Tests

According to the AEDA, 13 key odorants that were detected to have FD factor ≥2 were submitted to simulate the aroma of AMI using a recombination test with their measured concentration. As shown in Fig. 1, the AMI and recombination models were similar in aroma profiles, and no significant difference (*p* > .05) was observed in the eight sensory attributes between the AMI and recombination models (Fig. 1). The aroma of AMI was well reconstructed, indicating that these odorants have dominated contributions to the off-flavor. Furthermore, the result of the omission test showed that six compounds had noticeable effects on the sweaty, metallic, and plastic notes (Table 3). In short, the omission of tetramethylpyrazine, 2,4-di-*tert*-butylphenol, isovaleric acid, and 2-methylbutyric acid led to a significant decrease in the sweaty note. The omission of dodecanol and 2-acetylbutyrolactone led to notable decreases in the intensities of metallic and plastic notes, respectively. Taking all together, tetramethylpyrazine, 2,4-di-*tert*-butylphenol, isovaleric acid, and 2-methylbutyric acid were the key contributors to the sweaty notes; 2-acetylbutyrolactone and dodecanol were the contributors to the plastic and metal notes.

**Table 3.** Omission Tests of After Methanol Induction (AMI)

No.	Omission compound from AMIR	Sweaty	Deep-fried, Fatty	Metallic	Green	Roasted	Honey-like	Plastic	Floral
AMIR		4.10 ± 0.52	1.89 ± 1.21	2.47 ± 1.38	1.10 ± 1.61	0.77 ± 0.73	0.30 ± 0.18	2.44 ± 1.25	0.35 ± 0.20
1	3-Phenylpropionic acid	3.70 ± 0.29	1.90 ± 1.1	2.40 ± 0.43	1.00 ± 0.39	0.50 ± 0.38	0.40 ± 0.20	2.30 ± 0.33	0.30 ± 0.22
2	Nonanal	3.80 ± 0.24	1.90 ± 0.65	2.40 ± 0.42	0.90 ± 0.75	0.70 ± 0.35	0.40 ± 0.22	2.30 ± 0.73	0.40 ± 0.28
3	Methyl phenylacetate	4.00 ± 0.47	1.90 ± 1.00	2.30 ± 1.05	1.00 ± 0.40	0.70 ± 0.24	0.30 ± 0.16	2.30 ± 0.55	0.30 ± 0.22
4	Dodecanol	4.20 ± 0.20	2.00 ± 0.51	1.40 ± 0.32*	0.90 ± 0.56	0.60 ± 0.55	0.40 ± 0.25	2.20 ± 0.53	0.40 ± 0.33
5	Dimethyl phthalate	4.00 ± 0.28	1.90 ± 0.98	2.30 ± 0.50	0.90 ± 0.66	0.60 ± 0.30	0.40 ± 0.19	2.30 ± 0.32	0.40 ± 0.33
6	2-Acetylbutyrolactone	4.10 ± 0.24	1.90 ± 0.63	2.30 ± 0.84	1.00 ± 0.56	0.60 ± 0.44	0.50 ± 0.25	1.30 ± 0.56*	0.40 ± 0.30
7	Trimethylpyrazine	4.00 ± 0.38	1.90 ± 1.12	2.40 ± 0.62	0.90 ± 0.43	0.41 ± 0.23	0.60 ± 0.28	2.30 ± 0.56	0.20 ± 0.11
8	Phenethyl alcohol	4.20 ± 0.31	1.90 ± 0.90	2.40 ± 0.72	1.00 ± 0.48	0.60 ± 0.32	0.50 ± 0.21	2.30 ± 0.38	0.30 ± 0.24
9	2,6-Dimethylpyrazine	4.20 ± 0.33	1.90 ± 0.50	2.40 ± 0.78	0.90 ± 0.29	0.60 ± 0.30	0.40 ± 0.22	2.40 ± 0.62	0.30 ± 0.2
10	Tetramethypyrazine	3.30 ± 1.43*	2.00 ± 0.6	2.30 ± 0.72	0.90 ± 0.49	0.80 ± 0.30	0.60 ± 0.37*	2.20 ± 0.41	0.50 ± 0.23
11	2,4-Di-*tert*-butylphenol	3.00 ± 1.21*	1.90 ± 0.75	2.30 ± 0.49	0.90 ± 0.46	0.70 ± 0.16	0.40 ± 0.24	2.20 ± 0.50	0.30 ± 0.22
12	Isovaleric acid	2.80 ± 0.39*	2.00 ± 0.82	2.30 ± 0.67	0.80 ± 0.42	0.60 ± 0.35	0.60 ± 0.32	2.30 ± 0.78	0.30 ± 0.23
13	2-Methylbutyric acid	3.30 ± 1.15*	1.90 ± 0.93	2.30 ± 0.80	0.90 ± 0.48	0.80 ± 0.64	0.60 ± 0.34	2.00 ± 0.97	0.40 ± 0.25

*Significant (α ≤ 0.05).

## Discussion

The host system of *P. pastoris* has been developed to heterologously produce proteins (Yang & Zhang, 2018; Fischer & Glieder, 2019). In our recent research works, the *P. pastoris* GS115 strain has been used to express various heterologous proteins (Li et al., 2020a; Zhang et al., 2020a; Ni et al., 2021). In these processes, the fermented broth of *P. pastoris* GS115 was sniffed with a strong off-flavor that has negative effects on the aromatic quality of the proteins. Various compounds with off-flavor have been illustrated in yeast products by researchers. For instance, sulfide compounds [e.g. hydrogen sulfide (Dequin, 2001) and thiophene (Lee et al., 2000)], and phenolic substances (e.g., 2-/4-methoxyphenol) are commonly presented in beer and wine fermented by *S. cerevisiae*. Yeast extracts have been determined to contain various aromatic volatiles, including 2-/3-methylbutanoic acid, 2-/3-methylbutanal, 3-mercapto-2-pentanone, 4-hydroxy-2,5-dimethyl-3(2*H*)-furanone, 2,3-butanedione, 3-methylpyridine, 2-acetyl-1-pyrroline, phenylacetaldehyde, and 2-acetyl-2-thiazoline (Münch et al., 1997; Münch & Schieberle, 1998; Zhang et al., 2017). However, it has not been illustrated which compounds contribute to the off-flavor of the fermented broth with *P. pastoris* strains. In this study, the off-flavor was determined as an outstanding sweaty note and moderate metallic and plastic notes by the sensory evaluation. Furthermore, the GC–MS analysis, GC–O analysis, and omission test showed that the key sweaty odorants of *P. pastoris* were identified to be tetramethylpyrazine, 2,4-di-*tert*-butylphenol, isovaleric acid, and 2-methylbutyric acid. The key contributors to the plastic and metallic notes were identified to be 2-acetylbutyrolactone and dodecanol, respectively. Tetramethylpyrazine is reported in yeast to generate the fermented soybean-like notes (Raza et al., 2019). Isovaleric acid, which is the classical sweaty malodorant (Gross, 2007), brings the sweaty flavor to fish products (Fukami et al., 2006), mushrooms (Zhang et al., 2020b), alcoholic drinks (Lee et al., 2000), and milks (Fricke & Schieberle, 2020). Tetramethylpyrazine has a significant contribution to the overall aroma profile of food systems (Adams et al., 2008; Müller & Rappert, 2010). By comparison, it is clear that the sweaty-note contributors of *P. pastoris*, that is, tetramethylpyrazine, 2,4-di-*tert*-butylphenol, isovaleric acid, and 2-methylbutyric acid, were similar to those identified in other biological products. Interestingly, 2-acetylbutyrolactone and dodecanol were first identified to be the contributors to the plastic and metallic notes. Thus, this research will helppeople to understand the off-flavor of *P. pastoris* and other biological products.

*Saccharomyces cerevisiae* could generate higher alcohols such as isoamylol and amyl alcohol during wine and beer brewing (Ma et al., 2017) and could transform in downstream pathways to synthesize organic acids with unpleasant aroma, including isovaleric acid (Thierry et al., 2002) and 2-methyl butyric acid (Dickinson et al., 2000). It has been reported that higher alcohols and acids could be synthesized via the Ehrlich pathway from the degradation of amino acids, and the Harris pathway from glycometabolism (Avalos et al., 2013). The 2,4-di-*tert*-butylphenol is a common metabolite that has been reported in yeast and other organisms (Zhao et al., 2020) to provide a phenol note (Pang et al., 2021). In addition, it has been reported that *S. cerevisiae* synthesizes tetramethylpyrazine via valine metabolism and spontaneous reaction (Heidlas & Tressl, 1990; González et al., 2010). Potentially, tetramethylpyrazine, 2,4-di-*tert*-butylphenol, isovaleric acid, 2-methylbutyric acid, 2-acetylbutyrolactone, and dodecanol likely originate from a similar pathway of other microorganisms such as *S. cerevisiae*, but further studies are needed to confirm the putative hypothesis. It was found that *P. pastoris* cells have different metabolic regulation in the case of different carbon sources such as glycerol and methanol (Ren et al., 2003; Van Der Klei et al., 2006). In the glycerol growth phase, the intracellular metabolic pathways are mainly concentrated in oxidative phosphorylation, glycolysis, tricarboxylic acid cycle cycle, and electronic respiratory chain. During the methanol induction period, the intracellular metabolic pathway is mainly concentrated in the methanol metabolic pathway (Ren et al., 2003). In the glycerol growth phase, *P. pastoris* grows in a medium containing glycerol as a carbon source that represses the expression of methanol promoter AOX1 through transcription repressor factors such as Mig1, Mig2, and Nrg1, and hexose transporters such as Hxt1 and Hxt2. At this stage, the related induction of exogenous protein was strongly inhibited due to the carbon source metabolic repression (Orman et al., 2009; Vogl et al., 2018). In the methanol induction stage, methanol activates AOX1 transcriptional expression through transcriptional activators Mxr1 and Mitl, and initiates the downstream metabolic pathway (Vanz et al., 2012; Vogl & Glieder, 2013). In comparison to the AMI, the medium added with MAM and the fermented broth BMI have a much lower content of off-flavor contributors, that is, tetramethylpyrazine, 2,4-di-*tert*-butylphenol, isovaleric acid, 2-methylbutyric acid, 2-acetylbutyrolactone, and dodecanol (Table 1), indicating that these compounds were synthesized after the methanol induction. Thus, the synthesis of volatiles with the off-flavor might be related to the methanol induction pathway in relation to the AOX1 activations, providing a fundamental basis for regulating the biosynthetic pathway to control the off-flavor. However, further research is needed to investigate how the off-flavor contributors are synthesized after methanol induction. Additionally, the gene-editing technologies such as CRISPR–Cas9 can facilitate blocking the synthetic pathway of off-flavor contributors, based on the illustration of the off-flavor synthetic pathway in the *P. pastoris* strains.

## Data Availability

The datasets generated during and/or analyzed during the current study are available from the corresponding author on reasonable request. This article does not contain any studies with animals performed by any of the authors.
